# Tumour Cell Generation of Inducible Regulatory T-Cells in Multiple Myeloma Is Contact-Dependent and Antigen-Presenting Cell-Independent

**DOI:** 10.1371/journal.pone.0035981

**Published:** 2012-05-29

**Authors:** Sylvia Feyler, Gina B. Scott, Christopher Parrish, Sarah Jarmin, Paul Evans, Mike Short, Katherine McKinley, Peter J. Selby, Gordon Cook

**Affiliations:** 1 Transplant Immunology Group, Academic Department of Haematology and Oncology, University of Leeds, Leeds, United Kingdom; 2 Department of Haematology, Calderdale and Huddersfield NHS Trust, Huddersfield, United Kingdom; 3 Haematological Malignancy Diagnostic Service, Leeds Teaching Hospitals NHS Trust, Leeds, United Kingdom; 4 Department of Clinical Immunology, Leeds Teaching Hospitals Trust, Leeds, United Kingdom; 5 Academic Department of Haematology and Oncology, University of Leeds, Leeds, United Kingdom; 6 Department of Haematology, St James’s Institute of Oncology, Leeds Teaching Hospitals NHS Trust, Leeds, United Kingdom; New York University, United States of America

## Abstract

Regulatory T-cells (T_Reg_ cells) are increased in patients with multiple myeloma (MM). We investigated whether MM cells could generate and/or expand T_Reg_ cells as a method of immuno-surveillance avoidance. In an *in vitro* model, CD4^+^CD25^-^ *FoxP3* ^-^ T-cells co-cultured with malignant plasma cells (primary MM cells and cell lines) induced a significant generation of CD4^+^CD25^+^ *FoxP3* ^+^ inducible T_Reg_ cells (tT_Reg_ cells; p<0.0001), in a contact-dependent manner. tT_Reg_ cells were polyclonal, demonstrated a suppressive phenotype and phenotypically, demonstrated increased *FoxP3* (p = 0.0001), increased GITR (p<0.0001), increased PD1 (p = 0.003) and decreased CD62L (p = 0.007) expression compared with naturally occurring T_Reg_ cells. FACS-sorted tT_Reg_ cells differentiated into *FoxP* ^+^IL-17^+^ and *FoxP3* ^-^IL-17^+^ CD4^+^ cells upon TCR-mediated stimulation. Blocking experiments with anti-ICOS-L MoAb resulted in a significant inhibition of tT_Reg_ cell generation whereas both IL-10 & TGFβ blockade did not. MM tumour cells can directly generate functional T_Reg_ cells in a contact-dependent manner, mediated by ICOS/ICOS-L. These features suggest that tumour generation of T_Reg_ cells may contribute to evasion of immune surveillance by the host.

## Introduction

The paradoxical observation of tumor growth despite an attempt by the tumour-bearing host immune system to control and eliminate the malignant cells suggests that the anti-tumour immune response is being attenuated limiting competent immune surveillance (reviewed in [1]). This has been extensively studied by tumour immune-biologists, with results pointing towards soluble factors and altered antigenicity as mechanistic explanations. More recently, with the discovery of a number of different immune-regulatory cell types, focus has shifted towards cellular mediated tumour-induced immune suppression and evasion. Several different subsets of regulatory T-cells have now been identified including *naturally occurring* T_Reg_ cells (nT_Reg_ cells: CD4^+^CD25^+^ *FoxP3* ^+^), and inducible T_r1_ and T_H3_ CD4^+^T_Reg_ cells [2] as well as CD8^+^ T_Reg_ cells [3] and Double Negative T_Reg_ cells. Originally it was thought that T_Reg_ cells were centrally generated in the thymus, though more recently evidence suggests that peripheral generation is also possible, thereby providing a biological back-drop to investigating their role in the cancer-bearing host [4], [5]. In fact, several studies have shown that increased levels of T_Reg_ cells can be found in a variety of solid tumours [6], [7] and haematological malignancies [8], [9], [10].

Multiple Myeloma (MM), an incurable malignant plasma cell dyscrasia, is associated with both cellular and humoral immune deficiencies [11]. Many potential mediators of the immunologically hostile microenvironment have been proposed including tumour-derived TGFβ [12], Prostaglandin E_2_ (PGE_2_) and Interleukin-10 (IL-10) [13]. In addition to soluble mediators, we and others have demonstrated that T_Reg_ cell subsets are functional and increased in the peripheral blood of patients with MM, associated with their disease burden [14]. In particular, we demonstrated a higher level in the “pre-myelomatous” condition, MGUS but to a lesser extent than when full disease is present though higher levels of IL-10 were seen in the PB of MGUS compared with patients with MM. In light of this recent evidence, it would now seem that the most promising and synergistic approaches for cancer immunotherapy will be strategies that augment specific anti-tumor immunity whilst simultaneously reducing the effect of tumour-induced immune-regulation. However, in order to perform this later component, a greater understanding of the *in vivo* mechanism of tumour-induced immune suppression is needed.

In this study, using an *in vitro* model system, we demonstrate that the tumour cells of MM are not only capable of expanding nT_Reg_ cells but generating T_Reg_ cells *de novo*, mediated through cell contact. Through our experimentation, we demonstrate that surface ICOS-L on the tumour cells mediates this phenomenon and that the tumour-induced T_Reg_ cells whilst sharing some are phenotypic features also display phenotypic differences but are functionally similar to nT_Reg_ cells. The data presented here provides further evidence of direct tumour manipulation of the immune system to augment immune evasion and propagation of the malignant cell clone.

## Materials and Methods

### Cell Lines, Culture Medium and Reagents

The human MM cell lines (HMCL) U266B, KMS11, JIM3, JJN3 and RPMI8226 (in house) were expanded in mycoplasma-free cultures, maintained in complete tissue culture medium (CM: RPMI 1640 medium, 10% FCS, 2 mM L-Glutamine, 100 iu/ml penicillin, 100 µg/ml streptomycin) in ventilated tissue culture flasks at 37°Celsius in a 5% CO_2_ humidified incubator [15], [16], [17], . The HMCL used in co-culture were HLA class II positive (HLA-DR^+^), in particular expressing HLA-DR. HMCL were treated with 50 µg/ml Mitomycin C in CM for 30 minutes at 37°C, followed by 2 washes in CM, to limit tumour cell proliferation in the co-culture. Directly conjugated mAbs against CD4-APC, CD8-APC, CD3-PerCP, CD25-PE (all from BD Biosciences, Oxford, UK), *FoxP3*-FITC (eBioscience, San Diego, USA), ICOS-PE (BD clone DX29, BD Biosciences, Oxford, UK) and IL-17A-APC-A (eBioscience clone eBio64DEC17, eBioscience, San Diego, USA) were used according to the manufacturer’s protocol with corresponding isotype-matched controls.

Primary samples were obtained from patients with myeloma (n = 9) through iliac crest aspirations. The study was approved by the local ethics committee and written informed consent was obtained (NRES Committee Yorkshire & The Humber – Leeds East: Ref 04/Q1206/147). All samples were collected in sterile EDTA containers and mononuclear cells (MNC) were isolated by density gradient centrifugation on Lymphoprep (Axis-Shield, UK) and stored in foetal calf serum with 10% DMSO in the vapour phase of liquid nitrogen at -270°C until the day of analysis.

### In vitro Modeling of Tumour Cell and T-cell Interactions

Mononuclear cell (MNC) preparations were made from leukocyte concentrates provided by the National Blood Service. MNC were isolated by density gradient centrifugation on Lymphoprep (Axis-Shield, UK) and washed three times in PBS before use in the culture system. Peripheral blood lymphocyte (PBL) preparations were made by monocyte-depletion of the MNC fraction through plastic adherence by 2-hour incubation of MNC in CM at 37°C. Mitomycin C-treated HMCL were added to MNC/PBL with a responder:HMCL ratio of 2∶1 at 1×10^6^ PBMC/ml with MNC-only controls treated the same way. Supernatants were collected on day 7 of culture and frozen immediately at −80°C for later cytokine assessment. Cells were harvested on the same days for analysis by Flow cytometry.

### Cell Sorting and FACS Analysis

Four-colour flow cytometry was performed on a LSRII (BD Biosciences) and analysed with FACS DIVA software. Directly conjugated mAbs against CD4-APC, CD8-APC, CD3-PerCP, CD25-PE (all from BD Biosciences, Oxford, UK) and *FoxP3*-FITC (eBioscience, San Diego, USA) were used according to the manufacturer’s protocol with corresponding isotype-matched controls. 1×10^6^ cells were stained. The fixation and dead cell discrimination kit (Miltenyi Biotec, Bergisch-Gladbach, Germany) was used to exclude dead cells within the intracellular staining protocol. Using a sequential gating strategy, T_Reg_ cells were identified as CD4^+^CD25^+^ *FoxP3* ^+^ T-cells and expressed as a percentage of the CD4^+^ T-cell population. In order to perform functional analysis on the *in vitro* generated T_Reg_ cells, cells were pre-selected through magnetic cell separation by using the CD4 untouched method (Miltenyi Biotec, Bergisch-Gladbach, Germany) according to the manufacturer’s protocol and then FACS sorted using the surface antibodies CD4-APC, CD25-PE and CD127-Pacific Blue. Samples were sorted into a CD4^+^CD25^+^CD127^-^ T_Reg_ cells and CD4^+^CD25^-^CD127^+^ effector T-cells the MoFlo high performance multi-parameter cell sorter.

### Proliferation and Suppression Assays

MNC were sorted into CD4^+^CD25^-^ effector cells and CD4^+^CD25^+^ T_Reg_ cells as described above. The CD4^+^CD25^-^ responder cells were plated in 96 well round bottom plates (Nunc plates, Thermo Fisher Scientific, Roskilde, Denmark) in triplicates at a concentration of 1×10^5^ cells per well in CM. Purified CD4^+^CD25^+^ T_Reg_ cells were added at different concentrations (4∶1 and 8∶1 responder to suppressor ratio). The suppressive capability of the T_Reg_ cell fraction was determined by ^3^H-Thymidine incorporation for 18 hours at 1 µCi per well after 72 hours stimulation with CD3/CD28- Antibiotin MACSIbeads (Miltenyi Biotec, Bergisch-Gladbach, Germany) at a 1 bead: 2 cell concentration. ^3^H-thymidine incorporation in the stimulated responder only wells was set as 100% and a stimulation index (SI) calculated. Where indicated, cell populations were stained with 2 mM CFSE before co-culture and analysed by FACS.

### Th_17_ T-Cell Analysis

To assess if tumour-generated regulatory T-cells have the same capability to produce IL17-producing T-cells (Th_17_ cells) as nT_Reg_ cells, both populations were obtained on day 7 of co-culture with HMCL and day 0, respectively. Sorted cells populations were stimulated with anti-CD3 and anti-CD28 coated beads at a cell to bead ratio of 2∶1 and cultured for 5 days in CM at 1×10^6^ cells per ml. Six hours prior to intracellular staining, Brefeldin A (BFA) (10 µg/ml), Ionomycin (1 µM) and phorbol myristate acetate (PMA) (20 ng/ml) were added. Cells were then washed and stained as per intracellular staining protocols using the dead cell discriminator with CD4 Pacific Blue, *FoxP3* FITC and IL-17A AlexaFluor 647 (eBioscience, clone eBio64DEC17), using their corresponding isotypes as controls, analysed using the LSRII as described above.

### Cytokine Assessment

Capture and detection antibodies were used (BD Biosciences, Oxford, UK) according to the manufacturers protocol. In short, high protein binding 96 well ELISA plates (MaxiSorp, Scientific Laboratory Supplies Ltd., Hessle, UK) were coated at 4°C overnight with IL-10 and TGFβ capture antibodies at 1∶500 dilution in 1x 1 M NaHCO_3_ pH 8.2 at 100 µl per well. For TGFβ ELISA, serum samples were diluted 1∶5 with PBS and activated with 1N HCL at room temperature for 15 minutes and neutralized with 1N NaOH. After blocking with PBS containing 10% FCS for 2 hrs at room temperature, samples and standards were loaded at 100 µl per well and incubated at 4°C overnight. 100 µl per well detection antibody was then added at 1∶1000 dilution for IL-10 and 1∶500 dilution for TGFβ and incubated for 2 hrs at room temperature followed by Extravidin-Avidin conjugate (100 µl per well at 1∶500 dilution in PBS/Tween for 1 hr) and substrate solution (Sigma, Dorset, UK) for approximately 30 minutes for development in the dark. Samples were analysed in triplicate and measured spectrophotometrically at 405 nm. For LUMINEX Extracellular assay, spectrally encoded antibody-conjugated 5.6 µm polystyrene beads were used according to the manufacturer’s protocol. Plates were pre-wet and 25 µl antibody coated beads and 200 µl wash solution were added and washed once. Then, 50 µl incubation buffer was added to 100 µl standard or 50 µl sample/50 µl assay diluents. After a 2 hour incubation and washing, the plate was then incubated with 100 µl PBS with the cytokine specific biotinylated detector antibodies. The fluorescent streptavidin-RPE was added and after incubation was analysed with the Luminex IS software.

### T-cell Receptor Clonality by PCR

T-cell receptor (TCR) clonality was determined by PCR analyses of *TCRG* rearrangements as previously described [20]. In brief, DNA was isolated from FACS sorted cells and subjected to PCR performed using the BIOMED-2 multiplex strategy (InVivoScribe Technologies, San Diego, CA). PCR products were labeled in the 6FAM, HEX and NED fluorochromes and Vγ usage was identified using ABI Fluorescence detection. Positive controls for clonal T-cell populations were derived from the peripheral blood of patients with T-cell lymphoproliferative disease.

### Statistical Analysis

Results were analysed using SPSS version 14.0 for Windows software. Multiple independent variables were analysed with the Kruskal-Wallis test for non-parametric samples and with the Mann-Whitney-U test for 2 independent samples. A *p*-value of <0.05 was considered statistically significant. Comparison of patient samples was expressed as median values and co-culture experiments as mean values.

## Results

### Malignant Plasma Cells Induce Regulatory T-cell Generation

**Figure 1:**
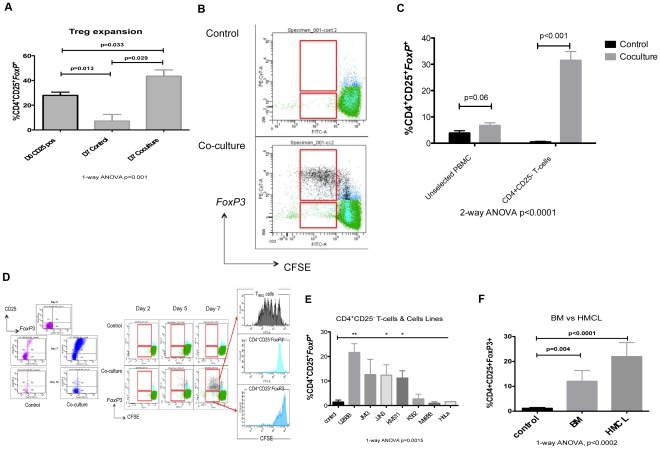
Regulatory T-cell induction by Myeloma tumour cells. A. The expansion of *FoxP3* ^+^CD25^+^CD4^+^ natural T_Reg_ cells, expressed as a percentage of CD4^+^T-cells. T_Reg_ cells were enumerated in the PB of healthy donors, after 7 days in CM and 7 days co-cultured with mitomycin C-treated U266B cells (n = 4). Results represent all experiments, expressed as mean ± SEM and analyzed using a 1-way ANOVA and student t-test. B. Expansion of nT_Reg_ cells when co-culture with HMCL results from cell division, illustrated by a representative flow cytometry plot of CFSE dilution. C. The generation of *FoxP3* ^+^CD25^+^CD4^+^ T-cells, expressed as a percentage of CD4^+^T-cells, in a co-culture assay with mitomycin C-treated U266B cells (n = 6) with varying starting populations: PB MNC, PBL CD25 depleted and CD4^+^CD25^-^ T-cells. Results demonstrate that increased generation of tumour-induced regulatory T-cells (tT_Reg_ cells) is seen with increasing purity of the seeded population. Results represent all experiments, expressed as mean ± SEM and analyzed using a 1-way ANOVA and student t-test. D. Representative flow cytometry plots demonstrating the generation of *FoxP3* ^+^CD25^+^CD4^+^ T-cells from CD4^+^CD25^-^ T-cells through cell division of de novo generated *FoxP3* ^+^ T-cells in a 7 day co-culture assay with mitomycin C-treated U266B cells. E. The generation of *FoxP3* ^+^CD25^+^CD4^+^ T-cells, expressed as a percentage of CD4^+^T-cells, in a co-culture assay of CD4^+^CD25^−^ T-cells (n = 10) with mitomycin C-treated MM cell lines (U266B, JJN3, JIM3 & KMS11), an erythro-leukaemia cell line (K562) and non-heamatopoietic cell lines (Mel888 & HeLa). Results represent all experiments, expressed as mean ± SEM and analyzed using a 1-way ANOVA and student t-test (**p<0.001, *p<0.01). F. The generation of *FoxP3* ^+^CD25^+^CD4^+^ T-cells, expressed as a percentage of CD4^+^T-cells, in a co-culture assay with fresh BM-derived myeloma plasma cells from patient samples (n = 7). Results demonstrate that increased generation of tumour-induced regulatory T-cells (tT_Reg_ cells) is seen with primary myeloma cells. Results represent all experiments, expressed as mean ± SEM and analyzed using a 1-way ANOVA and student t-test.

We have previously shown an increase in functional T_Reg_ cells in the peripheral blood of patients with MM, relating to the stage of their disease [14]. To examine the relationship between myeloma tumour cells and T_Reg_ cells, we first determined the effect of co-culturing naturally-occurring T_Reg_ cells (nT_Reg_ cells) with mitomycin-C treated HMCL (U266B HLA-class II^Pos^). When nT_Reg_ cells were sorted from the PB of healthy volunteers and cultured in CM alone, a significant reduction in the proportion of nT_Reg_ cells was observed (Day 0∶28.0%±2.6 vs Day 7∶7.3%±5.3; n = 4. p = 0.013). However, if co-cultured with HMCL, nT_Reg_ cell expansion was evident (Day 0∶28.0%±2.6 vs Day 7∶43.5%±5; n = 4, p = 0.029; 1-way ANOVA p = 0.001; Figure 1A and 1C). We hypothesized that the MM tumour cells could directly induce T_Reg_ cells, in the absence of antigen presenting cells. Firstly we examined the starting cell population in this model, culturing MNC and purified CD4^+^CD25^-^ effector T-cells (antigen-presenting cell free population) from healthy donor PB with mitomycin C-treated HMCLs. MNC from healthy controls contained a mean of 6.2%±0.4 CD4^+^ nT_Reg_ cells. nT_Reg_ cell depletion was very effective and achieved a CD25^+^CD4^+^ T cell contamination of 0.08%±0.05%, representing a >97.5% depletion efficiency (n = 7; p = 0.0001, Kruskal-Wallis test). Co-culture of unselected MNCs demonstrated a non-significant increase in T_Reg_ cells compared with controls (Day 0∶4.8%±1.1 vs Day 7∶7.6%±1.5; p = 0.06) which was enhanced when CD4^+^CD25^-^ T-cells only were seeded with cell lines (Day 0∶0.7%±0.3 vs Day 7∶31.6%±3.6; n = 10, p<0.001; Figure 1C & D).

Next to determine if this was a MM-specific effect, we co-cultured CD4^+^CD25^-^ T-cells with a selection of HMCL (U266, JJN3, JIM3 & KMS11), a myeloid-derived cell line (K562) and non-heamatopoietic cell lines (Mel888 & HeLa). A clear induction of T_Reg_ cells was seen with each of the HMCL and K562, but not the non-haematopoietic cell lines MEL888 or HeLa cell lines (n = 6, 1-way ANOVA p = 0.0015; Figure 1E). When sorted primary bone marrow plasma cells taken from patients with myeloma (n = 7) were co-cultured with CD4^+^CD25^-^ T-cells from healthy donors, a significant generation of T_Reg_ cells was seen (1.2%±0.31 vs 12.02±4.4, n = 7; p = 0.004), similar to the HMCL, U266B (1.2%±0.31 vs 21.9%±5.6, n = 7; p<0.0001; Figure 1F).

### Tumour-generated Regulatory T-cells are Phenotypically Different to Natural T_Reg_ Cells

**Figure 2:**
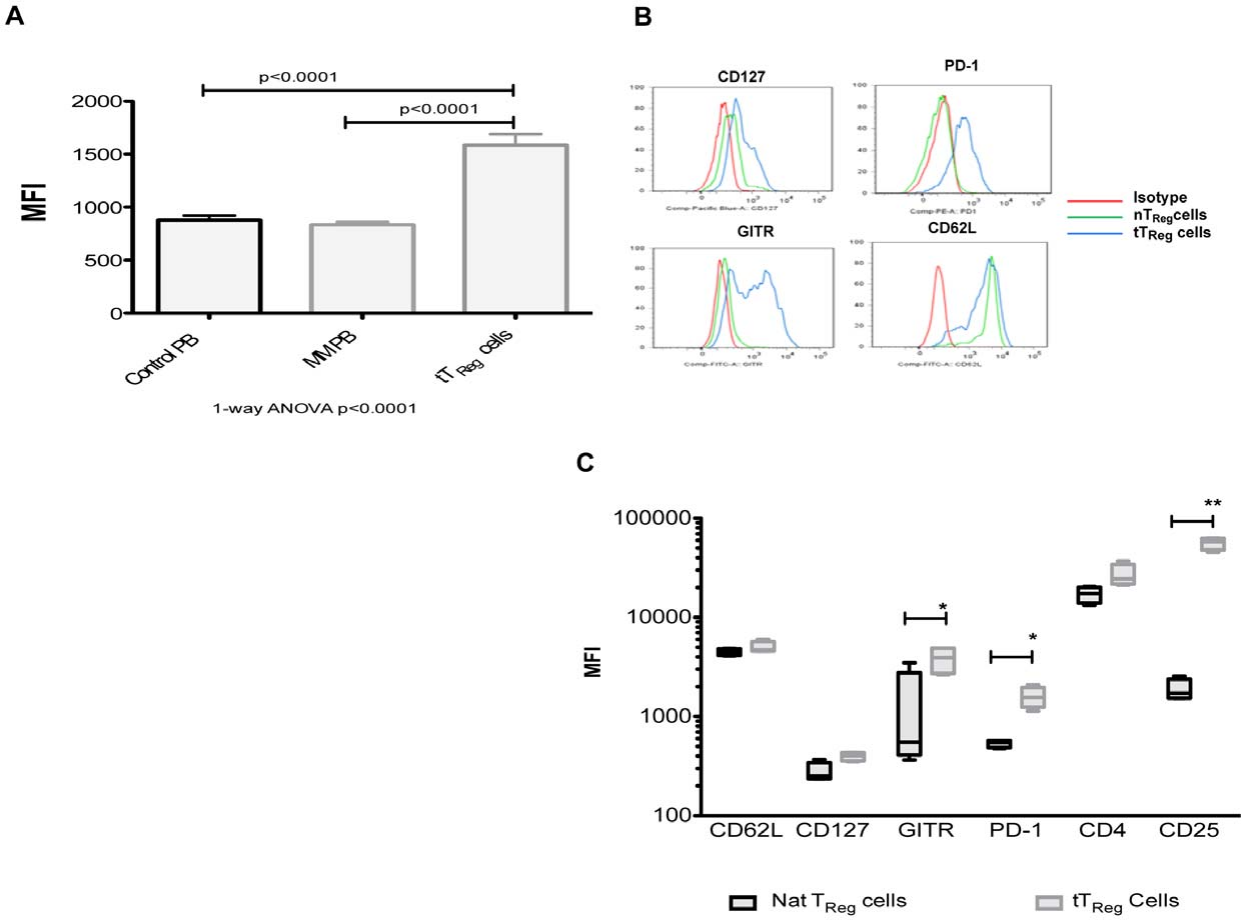
Phenotypic characteristics of tumour-induced regulatory T-cells. A. *FoxP3* expression, as determined by mean fluorescence intensity (MFI) in natural T_Reg_ cells from healthy controls (Control PB, n = 43), PB from patients with MM (MM PB, n = 43) and tT_Reg_ cells (n = 15) generated in co-culture from CD4^+^CD25^-^ T-cells (Kruskal-Wallis test). Results represent all experiments, expressed as mean ± SEM and analyzed using a 1-way ANOVA and student t-test. B. Representative histograms of naturally occurring T_Reg_ cell and tT_Reg_ cell surface expression of CD127, PD-1, GITR and CD62L. C. Summary of surface expression profiling of natural T_Reg_ cells and tT_Reg_ cells generated in co-culture, gated on *FoxP3* ^+^CD25^+^CD4^+^ T-cells (n = 4), expressed as mean fluorescence intensity (MFI). Results represent all experiments, expressed as mean ± SEM and analyzed using student t-test (*p<0.003, **p<0.0001).

Differences in phenotype between naturally occurring and inducible T_Reg_ cells have been reported [21], [22]. We therefore sought to characterize the phenotype of tT_Reg_ cells generated in our *in vitro* assay compared with naturally occurring T_Reg_ cells selected from steady PB of healthy volunteers. Given the potential for heterogeneity of response between the different samples from healthy volunteers, we utilized the one HMCL to provide consistency in the *in vitro* model, though similar results were generated using other MM cell lines (JIM3, JJN3 & RPMI8226- data not shown). When CD4^+^CD25^-^ T-cells were selected as the starting population, the level of *FoxP3* expression was significantly greater than naturally occurring T_Reg_ cells either from the PB of healthy controls or patients with MM (1585±101 vs 884±67,p<0.0001, Kruskal-Wallis test; Figure 2A). Next, using a sequential gating strategy, we examined the expression of key surface markers on CD4^+^CD25^+^ *FoxP3* ^+^ T-cells. tT_Reg_ cells demonstrated a similar level of CD127 (p = 0.413) and CD4 (p = 0.415) expression but demonstrated significantly higher levels of CD25 (43,492±6800 vs 1896±137,p<0.0001), GITR (70±5 vs 10±3,p<0.001) and PD-1 (49.8±9 vs 5.3±0.8,p = 0.003), as illustrated in Figure 2B and C. With regards to CD62L, there was an overall lower mean fluorescence intensity (MFI) compared to naturally occurring T_Reg_ cells (88.9±0.54 *vs* 97.3±0.54, p = 0.008; Figure 2C), but a bi-phasic pattern of expression suggests two populations of cells, some of which demonstrated similar expression of CD62L as naturally occurring T_Reg_ cells (Figure 2C). To determine the clonality of tumour-induced T_Reg_ cells, CD4^+^CD25^+^CD127^Dim^ T-cells were FACS sorted after 7 days of co-culture with mitomycin-C treated HMCL and DNA prepared from sorted cell populations. *TCRG* PCR was performed on genomic DNA derived from the tT_Reg_ cells. The spectrograph indicates multiple “spikes” representative of a polyclonal population in respect to the *TCRG* rearrangements, compared to a single “spike” representative of a monoclonal population (Figure S1).

### tT_Reg_ Cells though Functionally Similar to nT_Reg_ Cells Produce Interferonγ

**Figure 3:**
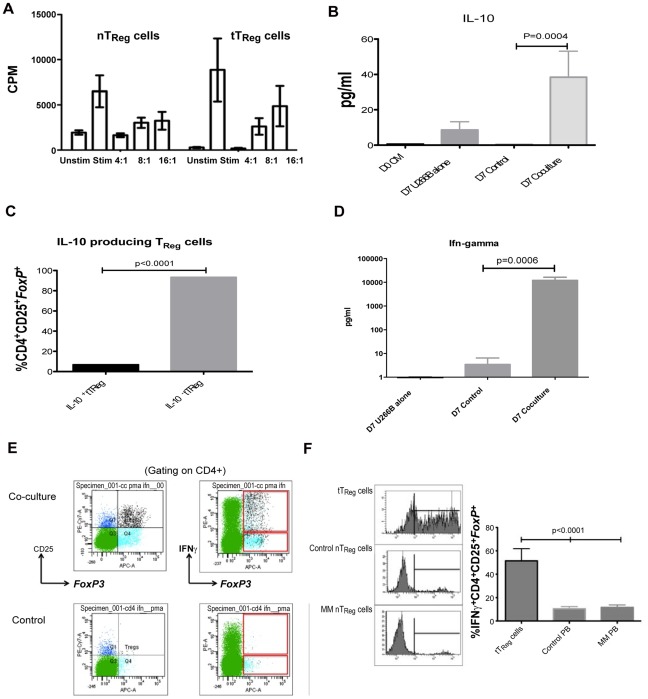
Functional characteristics of tumour-induced regulatory T-cells. A. Suppression of anti-CD3/anti-CD28-induced autologous T-cell proliferation by tumour-generated and naturally occurring T_Reg_ cells (n = 3), as determined by tritiated thymine incorporation. Results expressed as counts per minute (cpm)± SEM representing assays performed in triplicate. **Key:** Unstim – resting CD4^+^CD25^-^ T cells, Stim – CD3/CD28 stimulated CD4^+^CD25^-^ T-cells, 4∶1 etc – ratio of stimulated autologous T-cells to T_Reg_ cells. B. The generation of IL-10 in co-cultures of CD25^-^CD4^+^ sorted T-cells and HMCL, compared with HMCl alone and culture medium (n = 6, p = 0.0004). Results represent all experiments, expressed as mean±SEM and analyzed using student t-test. C. IL-10 production by tT_Reg_ cells after 7 days of co-cultures of CD25^-^CD4^+^ sorted T-cells and HMCL. Results represent all experiments, expressed as mean±SEM (n = 3) and analyzed using student t-test. D. IL-10 production by tT_Reg_ cells after 7 days of co-cultures of CD25^-^CD4^+^ sorted T-cells and HMCL. Results represent all experiments, expressed as mean±SEM (n = 3) and analyzed using student t-test. E. Representative flow cytometry plots demonstrating the generation of IFNγ^+^ *FoxP3* ^+^CD25^+^CD4^+^ T-cells from CD4^+^CD25^-^ T-cells in a 7 day co-culture assay with mitomycin C-treated U266B cells. F. The proportion of IFNγ-producing *FoxP3* ^+^CD25^+^CD4^+^ T-cells detectable in the peripheral blood of age-matched controls (n = 15), patients with MM (n = 15) and tTReg cells generated *in vitro* after 7 days of co-cultures of CD25^-^CD4^+^ sorted T-cells and HMCL (n = 3). Histograms represent IFNγ production by cells gated on *FoxP3*/CD25/CD4 positive staing. Results expressed as mean±SEM.

It has been reported that T_Reg_ cells from tumour-bearing hosts demonstrate altered suppressive capabilities [9], [23] though our studies in myeloma patients demonstrate that T_Reg_ cells are functionally active in suppression of autologous T-cell responses to TCR stimulation [14]. First we sought to determine the proliferative response of tT_Reg_ cells to TCR-mediated stimulation. CD4^+^CD25^-^ T-cells were isolated and co-cultured with HMCL for 7 days then CD4^+^CD25^+^CD127^Dim^ T-cells (tT_Reg_ cells) were FACS-sorted. tT_Reg_ cells were stimulated using CD3/CD28-coated beads for 5 days, determining their proliferative response by tritiated thymidine incorporation, comparing their response to sorted nT_Reg_ cells from healthy donors and patients with myeloma, similarly stimulated. tT_Reg_ cells demonstrated greater proliferative responses to TCR-mediated stimulation compared with nT_Reg_ cells from normal controls and MM patients, who demonstrated the weakest proliferative responses (16193±1860 cpm *vs* 1510±314 cpm *vs* 605±73 cpm, p<0.001; 1 way ANOVA). Next we examined their suppressive capabilities. tT_Reg_ cells generated in a 7 day co-culture were FACS-sorted and co-cultured with autologous T-cells stimulated with CD3/CD28-coated beads at the ratios described, for 5 days. The suppressive capacity of tT_Reg_ cells was compared with nT_Reg_ cells from healthy controls. We demonstrate that tT_Reg_ cells were able to suppress anti-CD3/anti-CD28-induced T-cell proliferation in a dose dependent fashion similar to naturally occurring T_Reg_ cells (Figure 3A). Next we sought to determine the cytokine production by tT_Reg_ cells in this culture system. When the supernatant was analysed for IL-10 on Day 7, the co-culture of T-cells with HMCL generated significantly higher levels of IL-10 compared to HMCLs or CD4^+^ CD25^-^ T-cells cultured alone (p<0.001; Figure 3B). However, when the production of IL-10 by tT_Reg_ cells was determined at the single-cell level by FACS, very few tT_Reg_ cells produced IL-10 (Figure 3C). When the culture supernatant was examined for the level of Interferonγ (IFNγ), the co-culture of T-cells with HMCL generated significantly higher levels of IFNγ compared to either HMCLs or CD4^+^CD25^-^ T-cells cultured alone (p<0.0006; Figure 3D). We sought to determine the cellular origin of IFNγ and demonstrated that IFNγ-producing tT_Reg_ cells could readily be identified, contributing to the production of IFNγ (Figure 3E). Analysis of nT_Reg_ cells from peripheral blood of healthy age-matched controls and patients with MM demonstrates a subset, albeit small subset, of nT_Reg_ cells that produce IFNγ (Figure 3F).

**Figure 4:**
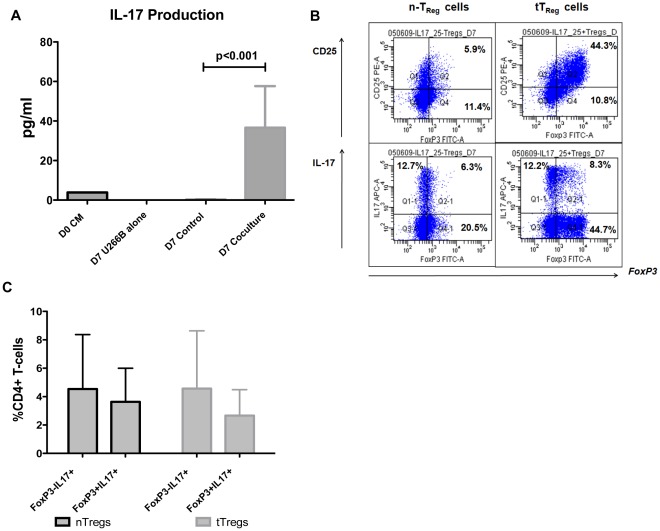
Natural and tumour-induced regulatory T-cell plasticity. A. The generation of IL-17 in co-culture supernatants of CD25^-^CD4^+^ sorted T-cells with HMCL, compared with HMCl alone and culture medium (n = 3). Results represent all experiments, expressed as mean±SEM. B. Representative dot-plots of IL-17 producing cells generated from re-stimulation of sorted tumour-generated and naturally occurring T_Reg_ cells after 5 days of re-stimulation. C. The proportion of IL-17 producing cells generated from re-stimulation of sorted tumour-generated and naturally occurring T_Reg_ cells, expressing *FoxP3* after 5 days of re-stimulation, expressed as a percentage of CD4^+^ T-cells. Results expressed as mean±SEM.

It is known that the effector T-cell lineage shows great plasticity and that human T_Reg_ cells can differentiate into IL-17-producing cells [24], [25]. When tT_Reg_ cells were generated in our *in vitro* culture model, a significant production of IL-17 was noted in the supernatant after 7 days of co-culture of CD4^+^CD25^-^ T-cells with HMCL (30±18 pg/ml vs 0.2±0.1 pg/ml; p<0.001; Figure 4A). Therefore, we wished to determine if Th17 cells could be generated directly from tT_Reg_ cells and thus, characterizing the plasticity of tT_Reg_ cells generated in our *in vitro* model, compared to naturally occurring T_Reg_ cells. CD4^+^CD25^-^ T-cells co-cultured with mitomycin-C-treated HMCL for 7 days were FACS-sorted and re-stimulated with CD3/CD28-coated beads with rhIL−2 20 U/ml for 5 days. For comparison, naturally occurring T_Reg_ cells were sorted using Miltenyi columns, co-cultured with mitomycin-C-treated HMCL for 7 days, then FACS-sorted and stimulated under identical conditions. After re-stimulation, a sub-population of IL-17-producing CD4^+^ T-cells was identified from the FACS-sorted tT_Reg_ cells, similar to nT_Reg_ cells (4.08%±2.0 of nT_Reg_ cells *vs* 3.62%±2.0 of nT_Reg_ cells, p = 0.87; Figure 4B). Closer analysis demonstrated that a smaller sub-population of *FoxP3* and IL-17 double positive cells were generated, in similar quantities from both nT_Reg_ cells and tT_Reg_ cells (3.6%±2.4 of nT_Reg_ cells vs 2.7%±1.8 of tT_Reg_ cells, p = 0.7; Figure 4C).

### Myeloma-generated Regulatory T-cells are Induced by Surface ICOS/ICOS-L Interactions not Tumour-derived TGFβ

**Figure 5:**
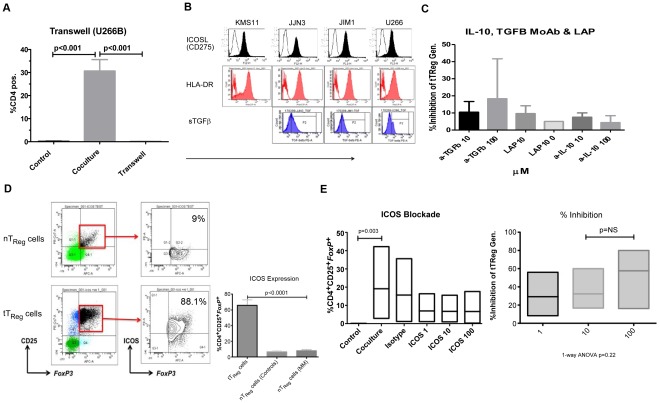
*In vitro* mechanisms of tumour regulatory T-cell induction. A. The generation of *FoxP3* ^+^CD25^+^CD4^+^ tT_Reg_ cells from CD4^+^CD25^-^ T-cells, expressed as a percentage of CD4^+^T-cells, in a co-culture assay with mitomycin C-treated U266 cells with and without transwell inserts (n = 7). Results represent all experiments, represented as mean±SEM and analysed using a student t-Test. B. Surface expression of HLA-DR, ICOSL (CD275) and TGFβ by human myeloma cell lines. C. Inhibition of tT_Reg_ cells generation from CD4^+^CD25^-^ T-cells by co-culture with HMCL (n = 3) through blockade of TGFβ and IL-10 using monoclonal antibodies and Latency-associated Peptide (LAP). Results represent all experiments, expressed as mean (±SEM). D. Surface expression of ICOS by T_Reg_ cells, presented as both percentage expression of CD4^+^CD25^+^ *FoxP3* ^+^ cells and representative dot plots. Results represent all experiments, expressed as mean ±SEM (n = 3) and analysed using a student t-test. E. Inhibition of tT_Reg_ cells generation from CD4^+^CD25^-^ T-cells by co-culture with HMCL through blockade of anti-ICOS-L (αICOS 1, 10, 100 µM) monoclonal antibody (n = 6), expressed as percentage of CD4^+^ T-cells and percent inhibition of tT_Reg_ cell generation. Results represent all experiments, illustrated as median with maximum and minimum values and analysed using a student t-test.

The mechanisms for controlling the induction and expansion of T_Reg_ cells remains to be fully clarified with some investigators demonstrating soluble factors as central to induction whilst others emphasize cell-to-cell contract, especially with dendritic cell contact, as key [26], [27], [28]. We adapted our antigen presenting cell-free *in vitro* model to investigate the role of humoral factors versus contact mediation. CD25^-^CD4^+^ T-cells were isolated from PB and co-cultured with mitomycin C-treated HMCL for 7 days with and without transwell separation. The generation of CD4^+^CD25^+^ *Foxp3* ^+^ T_Reg_ cells through co-culture with HMCL was significantly reduced by abolishing cell-to-cell contact (30.7%±5 CD4^+^ tT_Reg_ cells vs 0.11%±0.04 CD4^+^ tT_Reg_ cells, n = 7, p<0.001; Figure 5A). The inhibition of tumour-generated CD4^+^ T_Reg_ cells through abolition of cell-to-cell contact was associated with a reduction in the production of IL-10 (69.8±33.6 pg/ml *vs* 6.3±0.01 pg/ml, n = 3, p = 0.079) and IFNγ (12154±4174 pg/ml *vs* 0.04±0.01 pg/ml, n = 3, p<0.001).

The role that surface TGFβ plays in the induction and, in conjunction with IL-10, the propagation of T_Reg_ cells has been extensively studied in both human and murine systems [22]. We therefore wished to determine the role that TGFβ may play in the generation of tT_Reg_ cells in our model. Whilst HMCL produce soluble TGFβ (data not shown) they express the modulatory cytokine on their surface (Figure 5B) in addition to HLA class II (DR) and the negative co-stimulatory molecule (second signal) ICOSL (CD275). Therefore, CD4^+^ CD25^-^ T-cells were isolated from PB and co-cultured with mitomycin C-treated HMCL for 7 days with and without the specific TGFβ antagonist, Latency Associated Peptide (LAP) and an anti-TGFβ monoclonal antibody (MoAb). Neither LAP nor anti-TGFβ MoAb demonstrated significant inhibition of tT_Reg_ cell generation in our *in vitro* model (Inhibition with anti-TGFβ MoAb: 10.5%±6.3 at 10 µM and 18.4%±23 at 100 µM, n = 3; Inhibition with LAP: 9.6%±4.6 at 10 µM and 5.0%±0.1 at 100 µM, n = 3; Figure 5C Data). Similarly, the use of anti-IL-10 MoAb failed to demonstrate any significant inhibition of tT_Reg_ cell generation (7.5%±2.5 at 10 µM and 4.3%±4.1 at 100 µM, n = 3, Figure 5C).

The B7 family members, ICOS/ICOSL have previously been implicated in T_Reg_ cell generation. When examined, HMCL express surface ICOS-L (Figure 5B). We therefore determined the level of ICOS expression on newly generated tT_Reg_ cells and nT_Reg_ cells. A mean of 65.6%±7 tT_Reg_ cells expressed surface ICOS (n = 5) compared with 6.6%±1.5 nT_Reg_ cells from age-matched controls (n = 14) and 8.1%±1.3 nT_Reg_ cells from patients with MM (n = 10; p<0.0001; Figure 5D). Thus we added an anti-ICOS-L MoAb, in increasing concentrations, to CD4^+^CD25^-^ T-cells isolated from PB and co-cultured with mitomycin C-treated HMCL for 7 days. The anti-ICOSL was able to demonstrate a reduction in tT_Reg_ cell generation with an incremental inhibitory effect with increasing concentrations (Inhibition of tT_Reg_ cell generation: 29.2%±10 at μ1M, 32.3%±9.5 at 10 µM and 57.6%±14.5 at 100 µM, n = 6, Figure 5E).

## Discussion

The efficient generation of an immune response is coupled with a regulatory system to limit that response, preventing the destruction of healthy cells and tissues [29]. However, transformed malignant tissue may adopt one or more mechanisms to interfere with either the effector immune response or the regulatory cell compartment in an attempt to evade immune surveillance [30], [31], [32], [33], [34]. In myeloma, both dysfunctional effector responses and augmented regulatory cell compartments have been described [9], [12], [35], [36]. To date, the origin of the expanded regulatory T-cell population has remained elusive. We therefore sought to elucidate a causal relationship between the tumour cell in myeloma and T_Reg_ cell generation. Our model system demonstrates, for the first time, a direct induction of T_Reg_ cells (tT_Reg_ cells) by both fresh myeloma cells and cell lines that demonstrate the phenotype and functionality of T_Reg_ cells whilst inducing IL-10 production in non T_Reg_ cells. However, phenotypic differences between tT_Reg_ cells induced and naturally occurring T_Reg_ cells were noted. In particular tT_Reg_ cells demonstrated a CD25^High^ *FoxP3* ^High^GITR^+^PD-1^+^ phenotype, distinct from naturally occurring T_Reg_ cells. IFNγ-producing regulatory T cells have been described in the setting of intestinal infection and allograft rejection and evidence suggests a central role of IFNγ in inducible T_Reg_ cell generation [37], [38]. IFNγ cellular effects are mediated through STAT1 phoshorylation and it is known than there is a STAT1 binding site in the proximal region of the *FoxP3* gene promoter in humans (but not mice) [39], [40]. In addition to which, IFNγ has been shown to mediated *FoxP3* gene induction in synergy with IL-27[41]. Here we demonstrate for the first time in a cancer setting, the generation of inducible T_Reg_ cells from CD4+CD25- T cells where a subset produce IFNγ *in vitro*. In addition, similar to *naturally occurring* T_Reg_ cells, CD4^+^CD25^+^CD127^Low^ FACS sorted tT_Reg_ cells demonstrate lineage plasticity by differentiating into IL-17-producing T-cells following further TCR-mediated stimulation [24], [42]. Furthermore, data published to date, has demonstrated a central role for antigen presenting cells (APC) in the interactions of tumour cells and T_Reg_ cells. However, in our *in vitro* system, myeloma tumour cells generate and expand tT_Reg_ cells in an APC-free manner, that is directly inducing CD4^+^CD25^-^ T-cells. The importance of the generation of previously considered pro-inflammatory cytokines in the generation and or propagation of T_Reg_ cells in cancer remain to be elucidated.

In patients with cancer, T_Reg_ cells are continuously exposed to tumour antigen (TA), either directly or through the tumour micro-environment which in turn, results in high levels of ICOS expression as has been demonstrated in melanoma and prostate cancer [7], [43]. T_Reg_ cells generated in this environment produce high levels of IL-10, which mediates their suppressive capabilities, especially dendritic cell function [44]. We demonstrate with our *in vitro* model that in myeloma, ICOS-L^+^ tumour cells directly induce tT_Reg_ cell generation mediated in a contact-dependent manner, in the absence of antigen-presenting cells, which is inhibited significantly though not totally using anti-ICOSL monoclonal antibodies. This model of induction tT_Reg_ cell however, does not account for chronic antigen stimulation by the tumour-bearing host, nor does it this culture system take allowances of the effects of immunomoduatory drugs such as steroids and IMiDs (Thalidomide, Lenalidomide, Pomalidomide) which may account for differences in ICOS expression between tT_Reg_ cell and T_Reg_ cells from MM patients, a significant level of IL-10 is produced though this contributes minimally to the generation of tT_Reg_ cells (as evidenced by lack of inhibition through monoclonal antibody blockade). Furthermore, although IL-10 production by ICOS-induced T_Reg_ cells has been documented in both human and murine *in vitro* systems [45], [46], [47], the tT_Reg_ cells induced in our system did not produce IL-10.

The role of TGFβ in both the generation of T_Reg_ cells and in the mediation of their suppressive effects has been the subject of conflicting reports and may relate to the experimental design of *in vitro* systems used to study this relationship. In murine model systems, TGFβ-mediated *FoxP3* induction in naïve T-cells augmented by IL-2, produce T_Reg_ cells with a suppressive phenotype though are rendered hypo-responsive to TCR-mediated stimulation [4], [48], [49]. In contrast, other investigators have demonstrated TGFβ independence in both the generation and mediation of suppression [50], [51], [52]. Murine prostatic and renal cell cancer cells have been shown *in vitro* to generate T_Reg_ cells mediated through TGFβ. We have previously shown TGFβ to have a central role in myeloma-mediated effector cell dysfunction and is detected at high level in peripheral blood and bone marrow [12], [36]. However, the data from our *in vitro* model did not demonstrate a prominent role for TGFβ in the induction of tT_Reg_ cell generation, despite the production of TGFβ in co-culture supernatant (data not shown) and expressed on the surface of tumour cells.

Recent studies have suggested a close relationship between CD4^+^CD25^+^ *FoxP3* ^+^ T_Reg_ cells and pro-inflammatory IL-17-producing T helper cells (Th17) [53]. In our studies, we demonstrate that tT_Reg_ cells have a capacity, upon TCR-mediated stimulation to generate IL-17 producing T-cells, both CD4^+^ *FoxP3* ^+^ and CD4^+^ *FoxP3* ^-^ cells, indicative of a plasticity of the tT_Reg_ cells, similar to previous reports [53], [54], [55]. More recently, it has ben shown that different myeloid-derived cellular subsets (CD14^+^HLA-DR^Dim^ *vs* CD14^+^HLA-DR^+^) can induce both T_Reg_ cell and Th17 cells, with a recognized degree of plasticity [56]. However, our *in vitro* model system is APC-free and devoid of the proposed myeloid-derived cellular subsets. Though the tT_Reg_ cells were generated by co-culture with HMCL, in the absence of additional TCR-mediated stimulation, the plasticity we observed with these tT_Reg_ cells was purely upon TCR-mediated stimulation in the absence of HMCL and suggests an independent functional plasticity of tT_Reg_ cells.

In summary, our *in vitro* studies demonstrate that the tumour cells of Myeloma are capable of inducing T-cells with the phenotypic and functional characteristics of T_Reg_ cells, associated with the production of IL-10 and IFNγ The induction of T_Reg_ cells is mediated by cell-to-cell contact with the ICOS/ICOS-L system demonstrating a central role in the induction. The data presented here offers a better understanding of the immune evasion adopted by MM tumour cells offering a potential opportunity to manipulate the tumour-bearing host immune micro-environment pharmacologically. The pre-clinical data presented here offers a scientific basis for the development of suitable clinical research protocols to test this *in vivo*.

## Supporting Information

Figure S1 **DNA PCR analyses of** ***TCRG*** **rearrangements of FACS sorted tT_Reg_ cells performed using the BIOMED-2 multiplex strategy.** Representative example of 3 experiments. Positive control used was peripheral blood from a patient with T-cell lympho-proliferative disease.
(TIFF)
