# Supplementary figures and images for: Tumour Cell Generation of Inducible Regulatory T-Cells in Multiple Myeloma Is Contact-Dependent and Antigen-Presenting Cell-Independent
Source: PLoS One. 2012 May 29;7(5):e35981. doi: 10.1371/journal.pone.0035981 (PMC3362588; doi:10.1371/journal.pone.0035981)

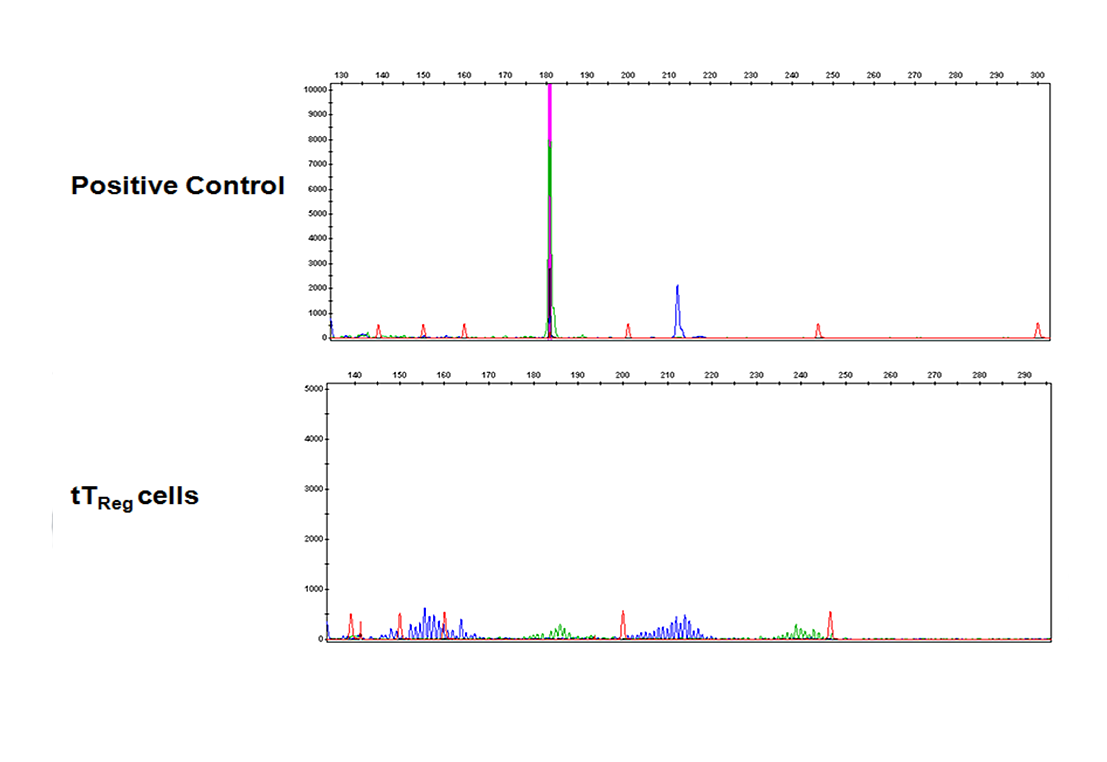

Supplement: Figure S1 — DNA PCR analyses of TCRG rearrangements of FACS sorted tTReg cells performed using the BIOMED-2 multiplex strategy. Representative example of 3 experiments. Positive control used was peripheral blood from a patient with T-cell lympho-proliferative disease. (TIFF) [file pone.0035981.s001.tif]
